# Nutritional status and postoperative recovery outcomes in patients undergoing orthopedic surgery

**DOI:** 10.3389/fnut.2026.1780584

**Published:** 2026-06-05

**Authors:** Dongmei Zhu, Xinyi Yin, Liangliang Qu

**Affiliations:** 1 Department of Orthopedics, The First Affiliated Hospital of Jinzhou Medical University, Jinzhou, China; 2 Department of Orthopedics, Jinzhou Medical University, Jinzhou, Liaoning, China; 3 Department of Orthopedics (Spinal Surgery, Trauma, and Joint Surgery), The First Affiliated Hospital of Jinzhou Medical University, Jinzhou, China

**Keywords:** functional outcomes, malnutrition, nutritional assessment, orthopedic surgery, postoperative recovery

## Abstract

**Background:**

Malnutrition is a prevalent yet often overlooked phenomenon among orthopedic patients, and it can adversely affect postoperative recovery and functional outcomes.

**Aim:**

To explore the relationships between preoperative nutritional status and postoperative recovery, complications, functional and healthcare utilization in the postoperative patients undergoing major orthopedic surgery.

**Methods:**

The retrospective cohort study was conducted at a tertiary orthopedic center and included adults who underwent major orthopedic surgery from January 2017 through December 2023. Preoperative nutritional assessment was conducted using the Malnutrition Universal Screening Tool, Nutritional Risk Screening 2002, anthropometric measurements, and laboratory parameters. The patients were classified into malnourished and well-nourished. Independent-samples *t*-tests and chi-square tests were used to compare postoperative outcomes.

**Results:**

Of 1,500 patients, 580 (38.7) were found to be malnourished. The older patients with malnutrition had a higher comorbidity and frailty score. They all exhibited a longer first ambulation time (44.6 vs. 29.4 h), less early mobilization (29.3% vs. 62.1%), a longer length of stay (10.9 vs. 7.0 days), and higher postoperative pain scores (all *p* < 0.001). Postoperative complications, such as surgical site infection, pneumonia, sepsis, and thromboembolic events, were very common in the malnourished group. There was poorer functional recovery, with lower Barthel Index scores at discharge and at 3 months, less independent mobility, and a decreased return to pre-injury activity. Malnourished patients were also more frequently readmitted within 30 and 90 days and consumed a higher amount of healthcare resources. Malnutrition was an independent predictor of delayed walking (aOR 2.84, 95%CI 2.21–3.65), extended hospitalization (aOR 3.12, 95%CI 2.45–3.97), and higher levels of post-operative pain (*β* = +1.08, 95%CI 0.92–1.24; all *p* < 0.001). It also contributed to the occurrence of various postoperative complications, including surgical-site infection (aOR 2.67), pneumonia (aOR 2.41), and sepsis (aOR 2.76). Functional rehabilitation became more difficult due to increased chances of a poor Barthel Index score at discharge (aOR 3.45).

**Conclusion:**

Preoperative malnutrition is significantly related to delayed recovery, more complications, worse functional outcomes, and increased healthcare use after orthopedic surgery. Timely nutritional screening and intervention could help enhance outcomes in the postoperative period.

## 1 Introduction

Orthopedic surgery is an important and expanding aspect of surgical care worldwide, especially for older persons requiring operations for degenerative joint disease, fractures, and musculoskeletal disorders that impair movements. Regardless of advancements in surgical procedures, anesthesia, and perioperative care, postoperative recovery after orthopedic surgery is still very diverse, with adverse effects, slow mobility, extended hospital stays, and decreased functionality continuing to present major clinical issues (1). Therefore, identifying controllable patient-linked risk factors that influence recovery pathways is critical to improving outcomes and optimizing healthcare resource use. Malnutrition is common among orthopedic patients upon hospital admission. Research carried out in hip fracture, trauma, and arthroplasty cohorts indicates that 15–40% of subjects are impoverished or at nutritional vulnerability (2). A greater occurrence has been reported among older individuals, those with fractures, and patients undergoing revision surgery (3). Age-associated physiological alterations, prolonged illness burden, systemic inflammation, and decreased dietary consumption after trauma or disease led to impaired nutritional status in this population.

From a pathophysiological perspective, malnutrition negatively impacts several systems that are crucial for the healing process following surgery. Collagen production, angiogenesis, and immune function are all hampered by protein-energy malnutrition and micronutrient depletion, thereby increasing vulnerability to infection and wound complications. According to Meermans et al. (4) and Hirsch et al. (5), a decline in muscle mass and power also leads to reduced mobility, slower rehabilitation, and diminished capacity to regain functional independence after surgery. These impacts are more pronounced in orthopedic patients, whose clinical outcomes are largely determined by prompt movement and physical recovery (4, 5).

A growing body of research indicates that poor nutritional condition is linked to increased incidence of postoperative sequelae, such as pneumonia, pressure injuries, thromboembolic problems, surgical site infections, and longer hospital stays following orthopedic surgeries. Serum albumin has been employed extensively in orthopedic outcome research and is the most frequently documented laboratory marker. Inadequate preoperative albumin concentrations have been linked to higher postoperative morbidity and mortality after fracture fixation procedures, total hip replacements, and total knee replacements (1, 6). In addition to albumin, orthopedic groups have employed validated nutritional assessment instruments, such as the Malnutrition Universal Screening Tool (MUST) and the Nutritional Risk Screening-2002 (NRS-2002). Research using these techniques has demonstrated that individuals classified as nutritionally at-risk had longer hospitalization, increased postoperative infection rates, and a higher chance of discharge to rehabilitation centers (7, 8). The relationship between dietary status and functional recovery outcomes has also been investigated. Malnutrition has been linked to prolonged mobilization, decreased independence in performing everyday tasks, and lower functional ratings at discharge and follow-up in individuals with hip fractures and orthopedic trauma (9, 10).

Thorough examinations of how preoperative nutritional status affects surgical recovery trajectories and clinical outcomes are certainly warranted, given aging orthopedic cohorts and substantial biological evidence linking nutrition to recovery. To enable the creation of multidisciplinary, nutrition-focused care pathways in orthopedic surgery, direct perioperative nutritional therapies, and risk assessment, it is imperative to fully understand these relationships. Thus, the goal of the current retrospective cohort study was to systematically assess the association between preoperative nutritional status and postoperative recovery outcomes in individuals undergoing orthopedic surgery. The present research aims to provide reliable evidence on the clinical consequences of malnutrition and to highlight nutrition as a critical, modifiable factor in optimizing orthopedic surgery outcomes by examining early recovery variables, postoperative complications, functional outcomes, and healthcare utilization.

## 2 Methodology

### 2.1 Study design and setting

A retrospective cohort study was conducted at a tertiary orthopedic care facility to assess the relationship between preoperative nutritional status and postoperative recovery outcomes. Health records of adults who underwent major orthopedic surgery between January 2017 and December 2023 were evaluated. This study was approved by the Ethics Committee of Jinzhou Medical University (approval number: JZMULL2025010).

### 2.2 Study population

Patients who were at least eighteen years old and had details of their preoperative nutritional assessment, perioperative care, and postoperative results were considered. Individuals who had pathological fractures associated with cancer, had an active systemic infection at hospitalization, or had inadequate laboratory, outcome, or demographic information were all excluded.

### 2.3 Nutritional status evaluation

Preoperative nutritional evaluation was performed within 48 h of hospitalization using a standardized multimodal framework. The Malnutrition Universal Screening Tool (MUST) and the Nutritional Risk Screening 2002 (NRS-2002) were used to assess individuals for nutritional risk. Body mass index and recorded inadvertent weight loss within the previous 6 months were among the anthropometric parameters. Biochemical data were derived from standard preoperative blood tests. Subjects were categorized as impoverished if one or more of the following requirements were met: serum albumin <3.5 g/dL, BMI < 18.5 kg/m^2^, unintentional weight loss >10%, MUST score ≥2, or NRS-2002 score ≥3. Individuals who did not meet these prerequisites were classified as well-nourished. Further laboratory indicators of nutritional reserves, inflammatory load, and immune competence, such as hemoglobin, total protein, prealbumin, total lymphocyte count, C-reactive protein, ferritin, vitamin D levels, neutrophil–lymphocyte ratio, and Prognostic Nutritional Index, were obtained for thorough characterization.

### 2.4 Baseline, functional, and perioperative variables

Baseline factors involved age, gender, BMI, smoking status, and recorded concurrent illnesses. Comorbidity incidence was measured using the Charlson Comorbidity Index, while frailty was evaluated using a standardized frailty score documented at enrollment. Functional status parameters included pre-fracture ambulatory ability, cognitive decline, residential status, admission within the previous year, and polypharmacy, defined as taking 5 or more medications. Perioperative information included the time from hospital admission to surgery, procedure duration, anesthesia modality, estimated blood loss, intraoperative transfusion, incidence of intraoperative hypotension, use of a cemented prosthesis, and postoperative admission to the critical care unit.

### 2.5 Postoperative outcomes

Postoperative outcomes were retrieved from hospital data and follow-up records. Time to initial ambulation, movement within 24 h, time to start oral feeding, postoperative pain ratings, duration of hospital stay, need for postoperative blood transfusion, and discharge disposition were all indicators of early recovery. Surgical site infection, wound dehiscence, pneumonia, urinary tract infection, deep vein thrombosis, pressure ulcers, cardiac events, and sepsis were among the postoperative issues reported. Readmission at 30 and 90 days, unscheduled emergency department visits, and revision surgeries within 90 days. Functional recovery factors included Barthel Index scores at discharge and at 3 months, achievement of complete load-bearing at 6 weeks, autonomous movement at 3 months, ongoing discomfort, restoration to pre-injury functional level, and discharge functional independence. Indicators of healthcare utilization included the length of rehabilitation, the number of outpatient visits, the need for home nursing care, referrals for community rehabilitation, caregiver reliance, and the overall length of follow-up.

### 2.6 Data handling

Missing data were assessed for all variables. Variables with <5% missingness were handled using complete case analysis, while variables with higher missingness were addressed using multiple imputation with chained equations under the assumption of missing at random. Sensitivity analyses were conducted to ensure consistency of results.

### 2.7 Statistical analysis

Continuous variables were presented as mean ± standard deviation and evaluated using independent-samples *t*-tests. Categorical parameters were displayed as frequencies and percentages and assessed employing chi-square tests. All statistical analyses were two-tailed, with *p*-values less than 0.05 considered statistically significant. Tests were carried out utilizing SPSS version 26.0. A multivariable analytical approach was used to assess the independent effect of nutritional status on outcome. The sensitivity analysis included the use of Definitions of malnutrition (albumin, NRS-2002, Prognostic Nutritional Index).

## 3 Results

### 3.1 Baseline demographic characteristics

The mean age of the malnourished group was significantly higher (72.5 ± 9.9 vs. 67.1 ± 10.4 years; *p* < 0.001), while the proportion of those aged ≥75 years was also greater (51.8% vs. 34.6%; *p* < 0.001). In terms of gender, the proportion of females was slightly higher in the malnourished group (62.5% vs. 58.2%), though this difference was not significant (*p* = 0.118). The mean BMI of the malnourished group was considerably lower compared to the well-nourished group (21.1 ± 3.3 vs. 25.9 ± 4.1 kg/m^2^; *p* < 0.001). The smoking status was similar between the two groups (24.6% vs. 21.2%; *p* = 0.173). On the contrary, the occurrence of diabetes mellitus (31.8% vs. 22.5%; *p* < 0.001), hypertension (55.7% vs. 48.9%; *p* = 0.012), and chronic kidney disease (16.2% vs. 9.3%; *p* < 0.001) was higher in the malnourished group (Table 1).

**Table 1:** Baseline demographic characteristics.

Parameter	Well-nourished (*n* = 920)	Malnourished (*n* = 580)	*p*-value
Age (years, mean ± SD)	67.1 ± 10.4	72.5 ± 9.9	<0.001
Female (%)	58.2	62.5	0.118
BMI (kg/m^2^, mean ± SD)	25.9 ± 4.1	21.1 ± 3.3	<0.001
Smoking (%)	21.2	24.6	0.173
Diabetes mellitus (%)	22.5	31.8	<0.001
Hypertension (%)	48.9	55.7	0.012
Chronic kidney disease (%)	9.3	16.2	<0.001

### 3.2 Core nutritional indicators

Albumin levels below 3.5 g/dL were observed in a higher number of cases in the malnourished group (66.1% vs. 9.2%; *p* < 0.001), and the average hemoglobin concentration was significantly lower (10.8 ± 1.9 vs. 12.9 ± 1.6 g/dL; *p* < 0.001). The malnourished patients had a MUST score ≥2 more often than the well-nourished patients (77.9% vs. 4.3%; *p* < 0.001) and an NRS-2002 score ≥3 more often (81.6% vs. 9.7%; *p* < 0.001). The incidence of unintentional weight loss of> 10% in 6 months was also higher among malnourished patients (53.4% vs. 6.1%; *p* < 0.001) and among those with BMI < 18.5 kg/m^2^ (28.1% vs. 3.1%; *p* < 0.001). Serum total protein levels below 6 g/dL were more prevalent in the malnourished group (48.5% vs. 7.9%; *p* < 0.001), and a greater number of patients were classified as ASA Class III–IV (45.8% vs. 28.9%; *p* < 0.001), indicating a higher physiological risk (Table 2).

**Table 2:** Core nutritional indicators.

Parameter	Well-nourished (*n* = 920)	Malnourished (*n* = 580)	*p*-value
Albumin <3.5 g/dL (%)	9.2	66.1	<0.001
Hemoglobin (g/dL, mean ± SD)	12.9 ± 1.6	10.8 ± 1.9	<0.001
MUST score ≥2 (%)	4.3	77.9	<0.001
NRS-2002 ≥ 3 (%)	9.7	81.6	<0.001
Weight loss ≥10% in 6 months (%)	6.1	53.4	<0.001
BMI < 18.5 (%)	3.1	28.1	<0.001
Serum total protein <6 g/dL (%)	7.9	48.5	<0.001
ASA class III–IV (%)	28.9	45.8	<0.001

### 3.3 Comorbidity and functional status

A Charlson Comorbidity Index ≥3 was more prevalent in the malnourished cohort (40.4% vs. 21.8%; *p* < 0.001), as was a frailty score ≥5 (38.9% vs. 17.6%; *p* < 0.001). Pre-fracture independent walking ability was less among the malnourished group (48.3% vs. 69.4%; *p* < 0.001), and cognitive impairment was more prevalent (18.5% vs. 9.3%; *p* < 0.001). A history of hospitalization in the previous year was more commonly reported among malnourished patients (32.9% vs. 18.7%; *p* < 0.001), as was a higher rate of nursing home residence (24.6% vs. 11.7%; *p* < 0.001). Use of multiple medications (≥5 drugs) was also seen more in malnourished patients (58.2% vs. 42.8%; *p* < 0.001), thus marking this group of patients as more difficult to manage clinically (Table 3).

**Table 3:** Comorbidity and functional status.

Parameter	Well-nourished (*n* = 920)	Malnourished (*n* = 580)	*p*-value
Charlson comorbidity index ≥3 (%)	21.8	40.4	<0.001
Frailty score ≥5 (%)	17.6	38.9	<0.001
Pre-fracture mobility independent (%)	69.4	48.3	<0.001
Cognitive impairment (%)	9.3	18.5	<0.001
Previous hospitalization <1 year (%)	18.7	32.9	<0.001
Residence in nursing facility (%)	11.7	24.6	<0.001
Polypharmacy ≥5 drugs (%)	42.8	58.2	<0.001

### 3.4 Characteristics of the perioperative period

The delay in surgery of more than 48 h was observed more often among the malnourished (38.1% vs. 23.9%; *p* < 0.001), and the operation time was longer (103.1 ± 29.2 vs. 94.7 ± 25.9 min; *p* < 0.001). Blood loss >500 mL was more frequent in the malnourished group (25.2% vs. 14.8%; *p* < 0.001), and this was associated with a higher rate of intraoperative transfusions (29.2% vs. 12.3%; *p* < 0.001). The use of general anesthesia was more prevalent in the malnourished group (61.5% vs. 48.1%; *p* < 0.001), whereas the proportion of patients receiving a cemented prosthesis did not differ significantly (46.8% vs. 41.5%; *p* = 0.067). The malnourished group had higher postoperative ICU admission rates (15.4% vs. 6.8%; *p* < 0.001) along with intraoperative hypotension (18.7% vs. 9.6%; *p* < 0.001). All these associations indicate that patients with malnutrition have a greater perioperative risk (Table 4).

**Table 4:** Perioperative characteristics.

Parameter	Well-nourished (*n* = 920)	Malnourished (*n* = 580)	*p*-value
Time to surgery >48 h (%)	23.9	38.1	<0.001
Operative duration (min)	94.7 ± 25.9	103.1 ± 29.2	<0.001
Estimated blood loss >500 mL (%)	14.8	25.2	<0.001
Intraoperative transfusion (%)	12.3	29.2	<0.001
General anesthesia (%)	48.1	61.5	<0.001
Cemented prosthesis (%)	41.5	46.8	0.067
ICU admission post-Op (%)	6.8	15.4	<0.001
Intraoperative hypotension (%)	9.6	18.7	<0.001

### 3.5 Outcomes of early postoperative recovery

**Figure 1:**
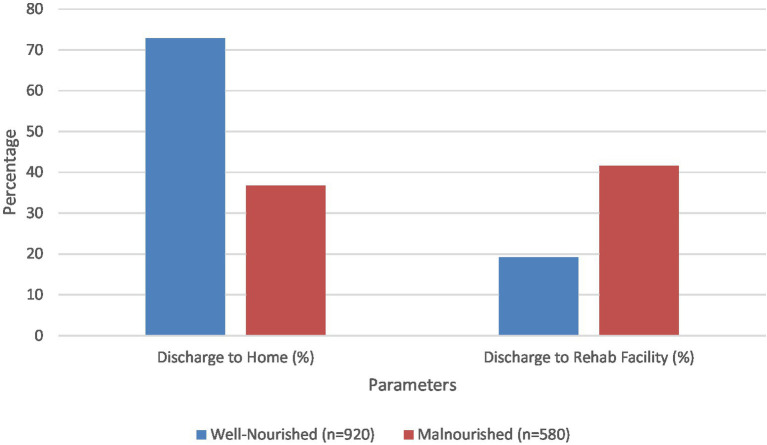
Early postoperative recovery outcomes.

The time to first mobilization in malnourished patients was longer (44.6 ± 12.5 vs. 29.4 ± 9.1 h; *p* < 0.001). Additionally, the percentage of patients who underwent early mobilization within 24 h was lower (29.3% vs. 62.1%; *p* < 0.001). Time to oral intake was delayed (18.9 ± 5.8 vs. 12.3 ± 4.0 h; *p* < 0.001), while length of hospital stay was prolonged (10.9 ± 4.2 vs. 7.0 ± 2.6 days; *p* < 0.001). Pain levels reported by malnourished patients were higher (5.1 ± 1.6 vs. 3.8 ± 1.2; *p* < 0.001), and the patients were transfused more often (22.7% vs. 8.6%; *p* < 0.001). The discharge distribution showed that fewer malnourished patients (36.8% vs. 72.9%; *p* < 0.001) returned home, while more patients were sent to a rehabilitation facility (41.6% vs. 19.1%; *p* < 0.001), which indicates that the functional recovery was delayed and post-discharge care requirements were higher (Table 5; Figure 1).

**Table 5:** Early postoperative recovery outcomes.

Parameter	Well-nourished (*n* = 920)	Malnourished (*n* = 580)	*p*-value
Time to first ambulation (hours)	29.4 ± 9.1	44.6 ± 12.5	<0.001
Early mobilization ≤24 h (%)	62.1	29.3	<0.001
Time to oral intake (hours)	12.3 ± 4.0	18.9 ± 5.8	<0.001
Length of stay (days)	7.0 ± 2.6	10.9 ± 4.2	<0.001
Post-Op pain score (0–10)	3.8 ± 1.2	5.1 ± 1.6	<0.001
Need for post-Op transfusion (%)	8.6	22.7	<0.001

### 3.6 Postoperative complications

The rate of surgical site infections was substantially higher among malnourished patients (11.7% against 3.5%; *p* < 0.001), while also, wound opening (5.1% vs. 1.1%; *p* < 0.001), lung infection (8.5% vs. 2.8%; *p* < 0.001), and unpredictable urinary passage (14.1% vs. 6.0%; *p* < 0.001). The occurrences of clots in the vein (6.8% vs. 2.6%; *p* < 0.001), bedsores (9.3% vs. 1.7%; *p* < 0.001), heart problems (8.9% vs. 3.3%; *p* < 0.001), and bloodstream infections (5.6% vs. 1.4%; *p* < 0.001) were all significantly greater in the malnourished group. Thus, the authors concluded that malnutrition remained an important factor in the development of various postoperative complications in the cohort studied (Table 6).

**Table 6:** Postoperative complications.

Parameter	Well-nourished (*n* = 920)	Malnourished (*n* = 580)	*p*-value
Surgical site infection (%)	3.5	11.7	<0.001
Wound dehiscence (%)	1.1	5.1	<0.001
Pneumonia (%)	2.8	8.5	<0.001
Urinary tract infection (%)	6.0	14.1	<0.001
Deep venous thrombosis (%)	2.6	6.8	<0.001
Pressure ulcer (%)	1.7	9.3	<0.001
Cardiac event (%)	3.3	8.9	<0.001
Sepsis (%)	1.4	5.6	<0.001

### 3.7 Readmission and reoperation outcomes

**Figure 2:**
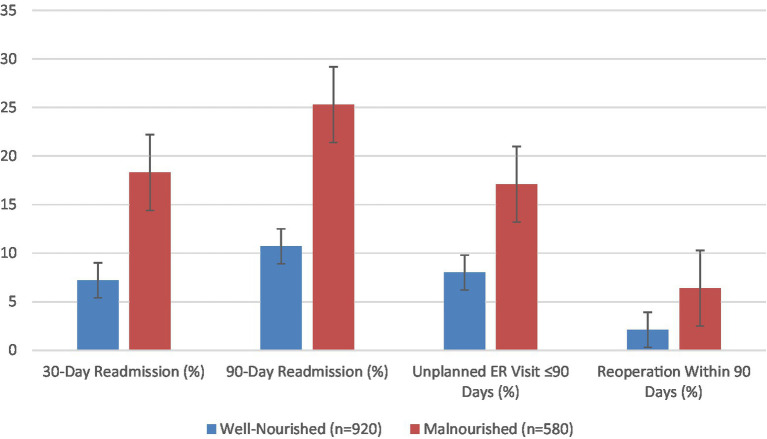
Readmission, reoperation, and mortality outcomes.

The malnourished patients, 30-day readmissions were considerably higher (18.3% vs. 7.2%; *p* < 0.001), as were 90-day readmissions (25.3% vs. 10.7%; *p* < 0.001) and unplanned ER visits within 90 days (17.1% vs. 8.0%; *p* < 0.001). Within 90 days, reoperation was also more prevalent in malnourished individuals (6.4% vs. 2.1%; *p* < 0.001). Such results gave evidence that malnutrition was a major factor leading to increased postoperative readmissions, repercussions, and even death among the targeted population (Figure 2).

### 3.8 Functional recovery outcomes

Malnourished patients had a significantly lower Barthel Index at discharge (63.5 ± 11.4 vs. 78.7 ± 10.2; *p* < 0.001), which remained lower at 3 months (71.4 ± 10.8 vs. 86.1 ± 9.7; *p* < 0.001) (Table 6). The percentage of patients reaching full weight-bearing at 6 weeks was lower in the malnourished cohort (38.5% vs. 69.2%; *p* < 0.001), and the same was observed for independent mobility at 3 months (41.2% vs. 71.9%; *p* < 0.001). Malnourished patients were more likely to report pain at 3 months (28.6% vs. 14.3%; *p* < 0.001) and less likely to return to pre-injury behavior (27.9% vs. 54.6%; *p* < 0.001). The discharge functional independence of malnourished patients (35.1% vs. 66.4%, *p* < 0.001) post-discharge, indicating slower recovery and lower post-discharge independence in this group, as shown in Table 7.

**Table 7:** Functional recovery outcomes.

Parameter	Well-nourished (*n* = 920)	Malnourished (*n* = 580)	*p*-value
Barthel index at discharge	78.7 ± 10.2	63.5 ± 11.4	<0.001
Barthel index at 3 months	86.1 ± 9.7	71.4 ± 10.8	<0.001
Full weight-bearing at 6 weeks (%)	69.2	38.5	<0.001
Independent mobility at 3 months (%)	71.9	41.2	<0.001
Persistent pain at 3 months (%)	14.3	28.6	<0.001
Return to pre-injury activity (%)	54.6	27.9	<0.001
Discharge functional independence (%)	66.4	35.1	<0.001

### 3.9 Advanced nutritional and immune markers

**Figure 3:**
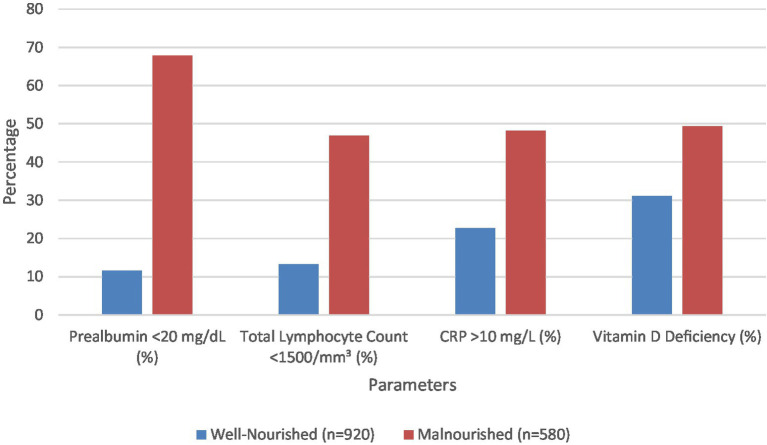
Advanced nutritional and immune markers.

Prealbumin <20 mg/dL was seen more often in malnourished patients (67.9% vs. 11.6%; *p* < 0.001), and so was total lymphocyte count <1,500/mm^3^ (46.9% vs. 13.3%; *p* < 0.001). Moreover, CRP elevation >10 mg/L among malnourished patients was more common (48.2% vs. 22.7%; *p* < 0.001), and the same was observed for indirect vitamin D deficiency (49.3% vs. 31.1%; *p* < 0.001). In addition, serum ferritin levels in the malnourished patients were marked (163 ± 57 vs. 118 ± 42 ng/mL; *p* < 0.001), and so was the neutrophil-lymphocyte ratio (4.8 ± 1.6 vs. 2.9 ± 1.1; *p* < 0.001). Last but not least, a Prognostic Nutritional Index <45 was more common among malnourished patients (39.6% vs. 8.4%; *p* < 0.001), indicating significant depletion of nutritional supply and immune support (Table 8; Figure 3).

**Table 8:** Advanced nutritional and immune markers.

Parameter	Well-nourished (*n* = 920)	Malnourished (*n* = 580)	*p*-value
Serum ferritin (ng/mL, mean ± SD)	118 ± 42	163 ± 57	<0.001
Neutrophil-lymphocyte ratio (mean ± SD)	2.9 ± 1.1	4.8 ± 1.6	<0.001
Prognostic nutritional index <45 (%)	8.4	39.6	<0.001

### 3.10 Healthcare utilization and economic outcomes

Malnourished patients had a longer rehabilitation duration (14.5 ± 4.8 vs. 9.2 ± 3.1 days; *p* < 0.001) and more outpatient visits over 6 months (3.4 ± 1.3 vs. 2.1 ± 1.0; *p* < 0.001). A larger proportion of malnourished patients required home nursing (39.2% vs. 18.6%; *p* < 0.001) and community rehabilitation referral (47.8% vs. 24.1%; *p* < 0.001). Also, dependence on informal caregivers was more frequent among malnourished patients (58.1% vs. 29.4%; *p* < 0.001). The malnourished group had a slightly shorter total follow-up time than the normal group (10.7 ± 3.8 vs. 11.2 ± 3.4 months; *p* = 0.041), which aligns with the higher post-discharge care needs and resource utilization observed in this group (Table 9).

**Table 9:** Healthcare utilization and economic.

Parameter	Well-nourished (*n* = 920)	Malnourished (*n* = 580)	*p*-value
Rehabilitation duration (days)	9.2 ± 3.1	14.5 ± 4.8	<0.001
Outpatient visits (6 months)	2.1 ± 1.0	3.4 ± 1.3	<0.001
Home nursing requirement (%)	18.6	39.2	<0.001
Community rehab referral (%)	24.1	47.8	<0.001
Informal caregiver dependence (%)	29.4	58.1	<0.001
Total follow-up duration (months)	11.2 ± 3.4	10.7 ± 3.8	0.041

### 3.11 Multivariable regression

Malnutrition significantly influenced delayed recovery, as evidenced by increased risks of delayed walking (aOR 2.84, 95% CI 2.21–3.65) and delayed swallowing (aOR 2.19, 95% CI 1.72–2.79). Furthermore, malnutrition increased the risk of a long hospital stay (more than 10 days) by almost 4 times (aOR 3.12, 95% CI 2.45–3.97). Finally, malnutrition increased postoperative pain intensity (*β* = +1.08, 95% CI: 0.92–1.24; all *p* < 0.001).

Regarding adverse outcomes, malnutrition independently increased the risk of surgical site infection (aOR 2.67), pneumonia (aOR 2.41), urinary tract infection (aOR 2.03), deep vein thrombosis (aOR 1.89), and sepsis (aOR 2.76). All estimates were statistically significant (*p* < 0.01).

Functional outcomes were considerably worse among malnourished patients. Malnutrition significantly increased the risk of a low functional state at discharge (Barthel Index <70): aOR, 3.45; 95% CI, 2.71–4.40. Moreover, inability to walk independently after 3 months and inability to perform activities typical before injury were both significantly associated with malnutrition: aOR 2.92 and aOR 2.88, respectively (Table 10).

**Table 10:** Multivariable regression analysis of nutritional status and postoperative outcomes.

Outcome	Adjusted measure	Effect estimate	95% CI	*p*-value
Early recovery outcomes				
Delayed ambulation (>24 h)	aOR	2.84	2.21–3.65	<0.001
Delayed oral intake (>24 h)	aOR	2.19	1.72–2.79	<0.001
Prolonged length of stay (>10 days)	aOR	3.12	2.45–3.97	<0.001
Postoperative pain score	β coefficient	+1.08	0.92–1.24	<0.001
Postoperative complications				
Surgical site infection	aOR	2.67	1.85–3.84	<0.001
Pneumonia	aOR	2.41	1.58–3.68	<0.001
Urinary tract infection	aOR	2.03	1.48–2.79	<0.001
Deep venous thrombosis	aOR	1.89	1.20–2.96	0.005
Sepsis	aOR	2.76	1.58–4.83	<0.001
Functional recovery outcomes				
Low Barthel index at discharge (<70)	aOR	3.45	2.71–4.40	<0.001
Failure of independent mobility (3 months)	aOR	2.92	2.30–3.70	<0.001
Persistent pain (3 months)	aOR	1.96	1.52–2.54	<0.001
Failure to return to activity	aOR	2.88	2.26–3.67	<0.001
Healthcare utilization				
Prolonged rehabilitation (>14 days)	aOR	2.74	2.12–3.53	<0.001
Home nursing requirement	aOR	2.58	2.01–3.30	<0.001
Caregiver dependence	aOR	2.91	2.30–3.69	<0.001

aOR, adjusted odds ratio; CI, confidence interval.

### 3.12 Sensitivity analysis

In the presence of alternative nutritional criteria, the relationship between malnutrition and SSI remained statistically significant, with a higher effect estimate for a Prognostic Nutritional Index below 45 (aOR = 3.12, 95% CI = 2.18–4.47). The subgroup analysis showed greater risks in older patients (age ≥75 years; aOR = 3.05), with a tripling of complication rates, and in frail patients (frailty ≥5), in whom the risk of poor postoperative ambulation increased significantly (aOR = 3.68). The removal of high-risk patients (Intensive Care Unit and ASA class IV) did not result in any notable reduction in the effect estimates. Similarly, the use of propensity score matching or multiple imputation modeling techniques yielded consistent results, indicating no potential for selection or missing-data bias in this study (Table 11).

**Table 11:** Sensitivity analyses for the association between malnutrition and outcomes.

Analysis type	Exposure definition	Outcome	Effect estimate (aOR)	95% CI	*p*-value
Primary model	Clinical malnutrition	Surgical site infection	2.67	1.85–3.84	<0.001
Alternative definition	Albumin <3.5 g/dL	Surgical site infection	2.89	2.01–4.15	<0.001
	NRS-2002 ≥ 3	Surgical site infection	2.74	1.92–3.91	<0.001
	PNI < 45	Surgical site infection	3.12	2.18–4.47	<0.001
Subgroup: age ≥75	Malnutrition	Complications (composite)	3.05	2.12–4.38	<0.001
Subgroup: frailty ≥5	Malnutrition	Poor mobility recovery	3.68	2.59–5.21	<0.001
Excluding ICU patients	Malnutrition	Complications	2.31	1.72–3.09	<0.001
Excluding ASA IV	Malnutrition	Functional decline	2.47	1.89–3.22	<0.001
Propensity score adjusted	Malnutrition	Any complication	2.21	1.74–2.80	<0.001
		Poor functional recovery	2.63	2.05–3.38	<0.001
Multiple imputation	Malnutrition	Complications	2.58	2.02–3.30	<0.001

## 4 Discussion

The results of the current retrospective cohort study show that malnutrition is strongly associated with poor baseline characteristics, compromised nutritional biomarkers, and suboptimal clinical parameters among patients undergoing orthopedic surgery. Malnourished patients were found to be much older, with lower BMI, with more disease burden, and with poorer biochemical nutrition indicators relative to well-nourished patients. These results highlight the importance of nutritional status before surgical intervention as a primary indicator of perioperative risk among orthopedic patients, and indicate that malnutrition may be more indicative of general physiological frailty than mere nutritional deficiency.

Old age was another significant contributor to malnutrition among our subjects, with over half of our malnourished patients being above the age of 75 years. This is supported by the idea that physiological deterioration associated with aging, such as sarcopenia, loss of appetite, and chronic inflammation, plays an important role in nutritional deterioration. As reported by Zink et al. (11), the development of multimorbidities and functional deterioration among elderly orthopedic patients is among the biggest contributors to nutritional impairment in these patients.

The nutritional markers showed a significant decline among patients with malnutrition, with notable reductions in BMI and an increased prevalence of underweight individuals (BMI < 18.5 kg/m^2^). This indicates protein-energy malnutrition and is consistent with Shi et al.’s findings (3), which revealed that low BMI was a common characteristic of frailty and poor tolerance for surgery among orthopedic cases (3). However, according to Zink et al. (11), BMI should not be relied upon solely to diagnose malnutrition among elderly patients, as indicated by their study (11).

Further biochemical disturbances were also identified in malnourished patients, specifically hypoalbuminemia, anemia, and decreased total serum protein concentration. Hypoalbuminemia was especially common and might be a result of insufficient dietary protein intake, ongoing inflammation, and other diseases, not just malnutrition itself. Research had previously demonstrated that hypoalbuminemia is a predictor of postoperative morbidity, leading to impaired wound healing and increased mortality in spinal and orthopedic surgeries (12, 13). Anemia may also indicate inadequate physiological reserves and impaired oxygen delivery, potentially hindering recovery. Overall, it appears that malnutrition is associated with biological vulnerability and poor resilience during surgery.

Indicators of nutritional risk, such as a MUST score > 2 and weight loss, were statistically associated with malnutrition. The results are consistent with current evidence recommending the use of validated screening tools to assess high-risk patients before surgery, in line with orthopedic recommendations (11). Furthermore, a higher ASA classification in malnourished patients (III-IV) indicates greater perioperative risk, in agreement with meta-analytical studies that show an association between malnutrition and surgical complications (14).

Malnutrition was also strongly linked to frailty, the Charlson Comorbidity Index, and functional disability. The commonality among these factors is likely due to shared pathophysiological processes, such as chronic inflammation, sarcopenia, and metabolic inefficiency. In addition, the decreased ability to walk independently and increased cognitive dysfunction in malnourished patients indicate that malnutrition may be part of a more complex syndrome than just poor nutrition. Studies on the relationship between malnutrition and frailty in orthopedic surgery patients show that both factors contribute to a worse outcome after surgery (3, 15).

Furthermore, malnourished individuals were noted to have more reliance on healthcare facilities before undergoing surgery, in that there was a high occurrence of prior hospitalization, living in nursing homes, and taking multiple medications. The use of multiple medications could worsen malnutrition by reducing appetite, affecting the digestive tract, and causing drug-nutrient interactions.

Additionally, perioperative outcomes were less desirable in malnourished patients, including more time taken before surgery, longer surgery times, greater blood loss, need for transfusions, use of general anesthetics, and intensive care unit admission. While this could result from decreased physiological reserves, a greater need for preoperative stabilization, and other factors, causality cannot be proven given the study design, and potential unmeasured confounders cannot be ruled out because of the association between malnutrition and disease severity and comorbidity.

Malnourished patients had a less favorable postoperative course, including delays in mobilization, decreased ability to consume oral food, elevated pain levels, and extended length of stay. All of these factors could be attributable to the known negative effects of malnutrition on wound healing, muscle strength, and inflammation, which together lead to reduced functional recovery (11). The higher pain levels found here may also be related to the inflammatory process.

Moreover, the discharge disposition confirmed the importance of malnutrition, with fewer direct discharges home and more admissions for rehabilitation or institutionalization. This implies long-term functional impairment, leading to greater demands for post-acute care. It is worth noting that similar results have been reported in prior orthopedic studies, in which poor nutritional status was linked to delayed recovery and readmissions (16−19).

Malnutrition was also found to increase the likelihood of postoperative complications, including surgical wound infection, wound dehiscence, pneumonia, UTIs, sepsis, thromboembolism, and pressure ulcers. These relationships are physiologically expected due to reduced immunity, impaired collagen synthesis, and decreased tissue oxygenation as consequences of malnutrition. Other meta-analyses have shown an increase in postoperative complication rates in both arthroplasty and spinal surgery patients who are malnourished (12, 17).

In addition, the malnourished group demonstrated higher incidences of readmissions, visits to the emergency department, and repeat surgical operations within both the 30-day and 90-day periods. This is probably due to delayed wound healing, persistent susceptibility to infections, and functional impairment following discharge, based on similar results from previous studies, where malnutrition has been independently associated with readmission and unfavorable surgical outcomes (13, 16, 19−23).

Malnourished patients showed significantly impaired functional recovery, evidenced by decreased Barthel index, delayed weight-bearing capacity, impaired mobility, and incomplete functional recovery compared to their baseline status. This result is expected, given prior studies showing that malnutrition and sarcopenia are predictors of limited rehabilitation potential (3, 21, 22, 24−26). Inflammatory and immune dysfunction can play a contributory role, as evidenced by increased CRP levels, NLR, and low PNI in the current study.

### 4.1 Strengths

• This is a retrospective cohort study with several strengths that enhance the credibility and depth of its findings.
• A large sample size of 1,500 patients over 7 years provides strong statistical power and improves the generalizability of results to tertiary orthopedic care settings.
• A multidimensional nutritional assessment approach was used, combining:
 o Standardized screening tools (MUST and NRS-2002).
 o Anthropometric measures.
 o Biochemical parameters.
 o Advanced inflammatory and immune markers. This comprehensive strategy allowed for more accurate stratification of malnutrition severity.
• The availability of complete baseline, functional, perioperative, and postoperative data enabled a detailed evaluation of the complex relationship between nutritional status and clinical outcomes.
• Functional recovery outcomes, post-discharge care requirements, and healthcare utilization were systematically assessed, allowing a broader understanding of the clinical and economic impact of malnutrition.
• The study provides an integrated, clinically relevant perspective on the role of nutritional status throughout the orthopedic recovery trajectory by capturing both early- and mid-term outcomes.

### 4.2 Limitations

- Due to its nature as a retrospective study, it may be hard to establish causality from the findings of the study.
- Because of the use of several nutritional measures, misclassification bias still could have occurred.
- The unmeasured factors like diet, socioeconomic status, and inflammatory disease activity may have affected the outcomes of the study.

### 4.3 Future recommendations

• Cost-effectiveness studies and healthcare resource utilization simulations are needed to inform policy decisions and support optimal allocation of limited healthcare resources.
• These analyses can help determine the economic value of:
 o Preoperative nutritional optimization programs.
 o Post-discharge support strategies.
 o Targeted perioperative interventions for high-risk patients.
• Preoperative nutritional assessment and optimization should be integrated into care pathways better to evaluate baseline nutritional status and its impact on outcomes.
• Longer follow-up periods (6 months to 1 year) are recommended to:
 o Assess long-term postoperative recovery.
 o Evaluate complication rates and functional independence.
 o Capture sustained effects of nutritional interventions.
• Inclusion of patient-reported outcome measures (PROMs) and quality-of-life assessments would provide a more comprehensive understanding of recovery trajectories.
• Such longitudinal and economic evaluations would enhance healthcare resource planning and improve predictive models for early identification of high-risk patients.
• These approaches would support the development of individualized perioperative care plans and more efficient allocation of post-discharge support services.

## 5 Conclusion

This paper has shown that preoperative malnutrition is a stronger predictor of poorer postoperative recovery. Complications, delayed functional recovery, and increased healthcare use in patients under caretakers going to orthopedic hospital stay, slow ambulation and oral intake, high postoperative pain scores, high readmission and re-operation rates, and increased reliance on caretakers compared to well-nourished patients: malnourished patients require timely detection and targeted treatment of nutritional deficiencies in orthopedic patients. The introduction of a systematic preoperative nutritional assessment and intervention may significantly affect surgical outcomes, accelerate functional recovery and patient independence, reduce healthcare expenditures, and decrease resource use following discharge, ultimately leading to an overall improvement in patient care.

## Data Availability

The raw data supporting the conclusions of this article will be made available by the authors, without undue reservation.
